# Generative artificial intelligence acceptance, anxiety, and behavioral intention in the middle east: a TAM-based structural equation modelling approach

**DOI:** 10.1186/s12912-025-03436-8

**Published:** 2025-07-01

**Authors:** Mona Gamal Mohamed, Polat Goktas, Shimaa Abdelrahim Khalaf, Aycan Kucukkaya, Ibrahim Al-Faouri, Ebtisam Abd Elazeem Saber Seleem, Awatef Ibraheem, Aya M. Abdelhafez, Saleh O. Abdullah, Hanan Nasef Zaki, Abdulqadir J. Nashwan

**Affiliations:** 1 Adult Health Nursing. RAK College of Nursing, RAK Medical and Health Sciences University, Al Qusaidat, Near RAK Hospital, PO Box: 11172, Ras Al-Khaimah, UAE; 2 UCD School of Computer Science, University College Dublin, Belfield, Dublin, Ireland; 3 College of Applied Medical Sciences in Yanbu Governorate, Taibah University, Yanbu, KSA Saudi Arabia; 4 Department of Community Health Nursing, Faculty of Nursing, Assiut University, Assiut, Egypt; 5 Institute of Graduate Studies, Istanbul University-Cerrahpasa, Istanbul, Turkey; 6 Dean of RAK College of Nursing, RAK Medical and Health Sciences University, Ras Al-Khaimah, UAE; 7 Jordan University of Science and Technology, Ar-Ramtha, Jordan; 8 Faculty of Nursing, Zarqa University, Zarqa, Jordan; 9 Nursing Administration Department, Assiut University, Assiut, Egypt; 10 Gynecological and Obstetric Nursing, Faculty of Nursing, Assiut University, Assiut, Egypt; 11 Department of Psychiatric and Mental Health Nursing, Faculty of Medicine & Health Science, Hodeidah University, Al Hudaydah, Yemen; 12 Psychiatric and Mental Health Nursing, Faculty of Nursing, Benha University, Benha, Egypt; 13 Nursing & Midwifery Research Department (NMRD), Hamad Medical Corporation, Doha, Qatar; 14 Department of Public Health, College of Health Sciences, QU Health, Qatar University, Doha, Qatar

**Keywords:** Artificial intelligence, Technology acceptance model, Nurse, Nursing education, Anxiety, Behavioral intention, Structural equation modeling

## Abstract

**Background:**

Adopting generative artificial intelligence (GenAI) in education rapidly transforms learning environments, yet nursing students’ acceptance and anxiety toward these technologies remain underexplored in Middle Eastern contexts. This study extends the Technology Acceptance Model (TAM) by incorporating constructs such as Facilitating Conditions (FC) and Social Influence (SI). It investigates the moderating role of Anxiety on Behavioral Intention to Use (BIU) generative AI tools.

**Methods:**

A cross-sectional study was conducted among 1,055 undergraduate nursing students across four Middle Eastern countries, including Egypt, Jordan, Saudi Arabia, and Yemen. Data were collected using a structured questionnaire comprising the Generative Artificial Intelligence Acceptance Scale and the Artificial Intelligence Anxiety Scale. Structural equation modeling was employed to evaluate relationships among Performance Expectancy (PE), Effort Expectancy (EE), FC, SI, and BIU, with Anxiety as a moderator. Descriptive statistics, confirmatory factor analysis, and path analysis were performed using SPSS and Python’s semopy library.

**Results:**

The model demonstrated strong explanatory power, with 75.09% of the variance in BIU explained by the TAM constructs and Anxiety. Path coefficients revealed significant positive relationships between PE (β = 0.477, *p* < 0.001), EE (β = 0.293, *p* < 0.001), FC (β = 0.189, *p* < 0.001), and SI (β = 0.308, *p* < 0.001) and BIU. Anxiety had the strongest moderating effect (β = 0.552, *p* < 0.001), indicating its critical role in shaping behavioral intentions. Gender, year of study, and access to technology emerged as significant demographic variables influencing acceptance and anxiety levels.

**Conclusions:**

This study emphasizes the importance of reducing anxiety and enhancing support systems to foster GenAI acceptance among nursing students. The findings provide actionable insights for designing culturally tailored educational interventions to promote the effective integration of AI in nursing education.

**Clinical trial number:**

Not applicable.

## Introduction

The rapid advancement of Generative Artificial Intelligence (GenAI) is reshaping the landscape of healthcare education and practice. Understanding the factors influencing its adoption among future healthcare professionals is crucial for leveraging its full potential. The Technology Acceptance Model (TAM), originally developed by Davis (1989) [1], has long served as a robust framework for examining user acceptance of emerging technologies. At its core, TAM posits that Perceived Usefulness (PU) and Perceived Ease of Use (PEOU) drive users’ Behavioral Intention to Use (BIU) a given technology. Previous research suggests that the explanatory power of the TAM significantly improves when relevant external variables are integrated into the model [2]. Additionally, perceived ease of use positively influences perceived usefulness, which in turn strengthens users’ intention to continue using a technology—ultimately facilitating its actual adoption [3, 4]. Over the past five decades, numerous technology adoption theories and acceptance models have been developed, refined, and extended to improve their predictive validity. These frameworks have been widely applied to evaluate the adoption of various Information and Communication Technology tools and services across diverse contexts [5, 6].

In the context of the Middle East, artificial intelligence (AI) adoption varies widely across countries due to disparities in digital infrastructure, policy, and education. Nations like the United Arab Emirates (UAE) and Saudi Arabia have made significant efforts in AI integration through national strategies and investments [7]. The UAE, for example, launched the National AI Strategy 2031, aiming to integrate AI into key industries such as healthcare, education, and finance [8]. Saudi Arabia’s Vision 2030 has also prioritized AI-driven technologies, investing billions in AI research, digital infrastructure, and governance [9]. However, countries such as Egypt, Jordan, and Lebanon demonstrate comparatively lower rates of AI adoption, largely attributed to challenges such as digital illiteracy, the absence of comprehensive AI regulations, and persistent socio-economic constraints [10, 11].

The adoption of AI technologies across Middle Eastern countries is shaped by a range of socio-cultural, economic, and infrastructural differences. While nations such as the UAE and Saudi Arabia have made notable progress in integrating AI into governance and healthcare systems, others like Egypt and Jordan continue to face barriers, including lower levels of AI literacy and limited access to digital infrastructure. For instance, although many students in Egypt report having some AI experience, the comparatively low rate of personal computer usage presents a significant obstacle to broader technology adoption. Yemen, meanwhile, encounters even more profound challenges—economic instability, inadequate AI education, and unreliable internet connectivity collectively pose substantial barriers to the effective implementation of AI technologies [12].

Recent studies have extended by the TAM by incorporating psychological and socio-cultural variables such as trust, AI-related anxiety, perceived risk, and ethical concerns [13]. Anxiety toward GenAI often stems from fears of job displacement, data privacy breaches, and algorithmic bias, all of which significantly affect user acceptance [14]. In countries like Kuwait and Bahrain, where AI is increasingly integrated into financial and educational sectors, institutional trust and regulatory frameworks positively influence behavioral intention [15]. In contrast, nations such as Palestine and Yemen struggle with economic instability and limited AI literacy, presenting significant barriers to AI adoption.

Socio-cultural factors also shape AI acceptance across the Middle East. Religious perspectives, particularly Islamic views on AI ethics and human-AI interaction, play a substantial role in influencing public attitudes, especially in countries with strong religious governance [16]. Gender disparities further affect engagement with AI, as women often face societal barriers and underrepresentation in technology fields [17]. However, targeted initiatives such as Saudi Arabia’s “Women in AI” programs aim to address these challenges by promoting AI education and employment opportunities for women [18]. As healthcare education continues to evolve, the integration of AI and machine learning represents a transformative milestone, with recent advancements demonstrating their growing role in shaping both educational practices and clinical applications [19, 20].

The successful adoption of novel technologies is shaped by a combination of technical, social, cultural, and psychological aspects that influence user attitudes and behaviors. To capture these complexities, frameworks such as the TAM and the Unified Theory of Acceptance and Use of Technology (UTAUT) have been widely employed. These models provide structured approaches to understanding the dynamics behind technology acceptance. Among them, TAM remains particularly popular due to its simplicity, validity, and effectiveness in identifying key drivers of technology adoption [19]. To this end, we extended the TAM by including Facilitating Conditions (FC) and Social Influence (SI) as external factors and incorporated Anxiety as a moderating variable. This study addresses a research gap by investigating nursing students’ acceptance of and anxiety toward GenAI tools across four Middle Eastern countries: Egypt, Jordan, Saudi Arabia, and Yemen. While previous studies have expanded TAM by integrating constructs such as trust, perceived risk, and ethical concerns [13–16], few have considered emotional variables like AI$\:-$related anxiety, especially in the context of healthcare education. By contextualizing TAM with socio-cultural and emotional dimensions, our study offers a novel contribution to understanding the multifaceted factors influencing GenAI adoption in nursing education across diverse regional settings.

## Methods

### Hypothesis model

This study investigates nursing students’ acceptance and anxiety toward Gen AI by extending the TAM. The model incorporates the core TAM constructs—PU and PEOU—through two sub-scales: Performance Expectancy (PE) and Effort Expectancy (EE), respectively. To maintain terminological consistency throughout the manuscript, we adopt the UTAUT-based labels PE and EE in all subsequent sections. Additional external factors, FC and SI are included to evaluate their impact on BIU Gen AI tools. Anxiety is introduced as a moderating variable, potentially influencing the relationships between these factors and behavioral intention. Previous research indicates that students’ perceptions of usefulness, ease of use, available support, and social encouragement can significantly impact their intention to adopt AI technologies [21–24]. Furthermore, technology-related anxiety may dampen the positive effects of these factors, limiting adoption.

To focus the analysis, the following hypotheses are proposed:

- **H1**: PE (aligned with PU) positively influences BIU GenAI tools.
- **H2**: EE (aligned with PEOU) positively influences BIU GenAI tools.
- **H3**: Anxiety moderates the relationships between PE, EE, and BIU, weakening their effects.

**Fig. 1:**
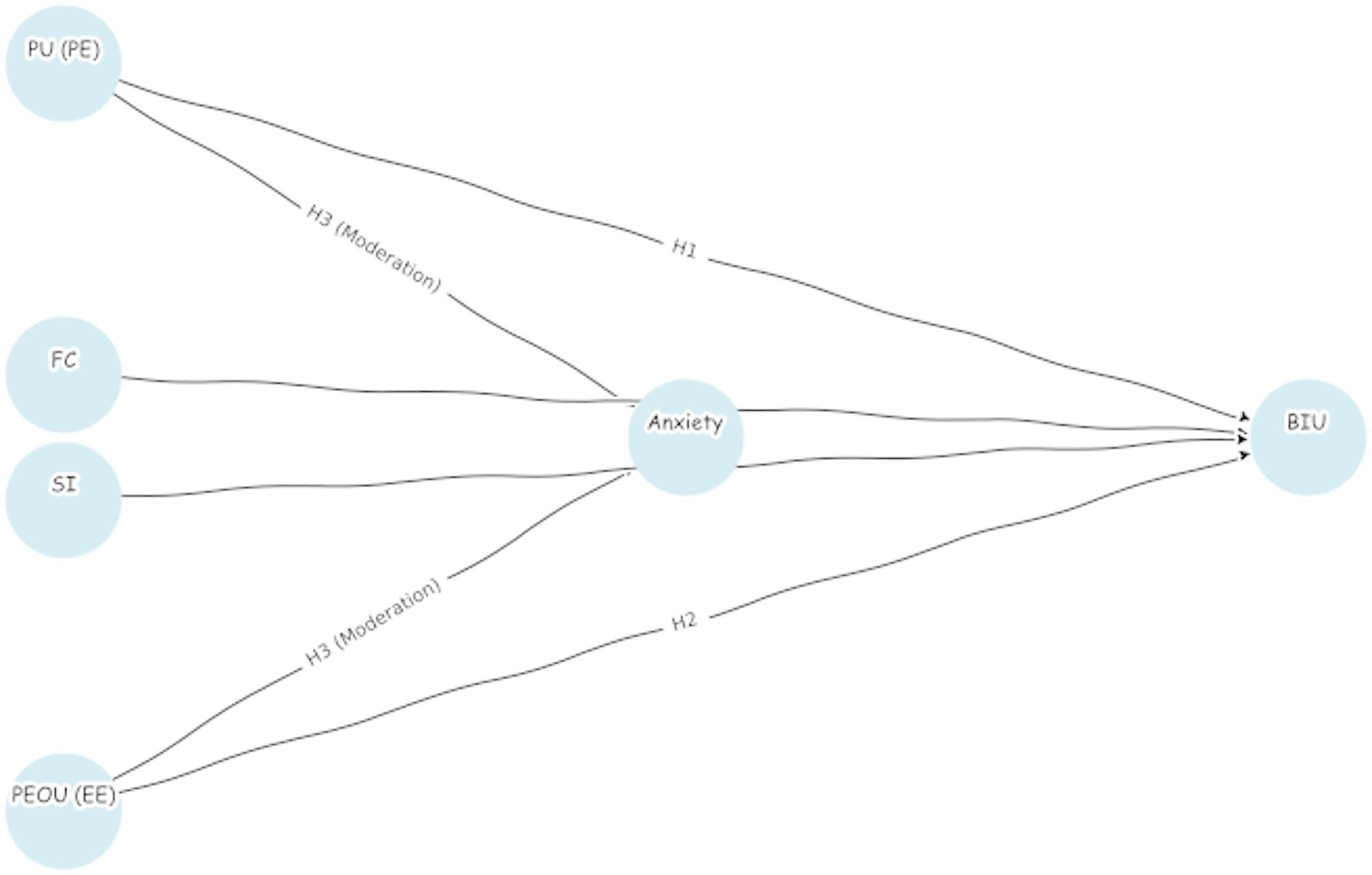
Hypothesis model

The hypothetical model is presented in Fig. 1, illustrating the direct relationships among the variables. This framework prioritizes key TAM constructs and their interaction with Anxiety, providing a clear basis for examining nursing students’ acceptance of GenAI technologies.

### Participants and procedures

A survey was designed to examine nursing students’ acceptance of and anxiety toward Gen AI across four Middle Eastern countries: Egypt, Jordan, Saudi Arabia, and Yemen. These countries were selected to represent a range of economic and educational environments with diverse levels of AI integration in nursing curricula. Institutions were chosen based on their accreditation status, the presence of structured nursing programs, and their willingness to participate in the study. To ensure diversity, we included both public and private institutions, as well as universities from different regions within each country.

Participants were recruited using a convenience sampling approach through institutional collaborations. Faculty coordinators at each university helped distribute the survey to nursing students via academic portals and official student groups. Participation was voluntary, and no incentives were provided, reducing potential coercion and aligning with ethical research practices. The study aimed to capture perspectives from students across different academic years, ensuring representation from those with varying levels of clinical exposure and technological familiarity.

A total of 312 students from Egypt, 287 from Jordan, 223 from Saudi Arabia, and 233 from Yemen participated in the survey, yielding the following estimated response rates: 62.4% for Egypt, 57.4% for Jordan, 55.8% for Saudi Arabia, and 58.3% for Yemen, based on the number of eligible students enrolled in the nursing programs at the time of data collection. Students were recruited from one accredited university in each country: Assiut University (Egypt), University of Zarqa (Jordan), Taibah University (Saudi Arabia), and Hodeidah University (Yemen). These institutions were selected for their national accreditation, strong nursing programs, and diverse student populations, making them representative of nursing education in their respective countries.

Students from all four academic years were invited to participate to ensure an even distribution across preclinical and clinical phases. While some institutions have integrated AI-related topics into their coursework, others provide AI exposure mainly through clinical training or informal learning. This variation allowed for a comprehensive assessment of Gen AI acceptance and anxiety among students with different levels of familiarity.

#### Sample size and recruitment

The sample size for each country was determined following the recommended practices for structural equation modeling (SEM), which suggest at least 20 participants per item on the scale to ensure robust analysis, with an additional 15% added to account for potential non-response bias. This approach aligns with the guidelines for sample adequacy in SEM, as outlined by Memon. et al. [25]. The questionnaire used in this study has a total of 43 dimensions, which include:

- 20 items in the *Generative AI Acceptance Scale (GAIAS)*,
- 23 items in the *Artificial Intelligence Anxiety Scale (AIAS)*.

The recommended sample size was calculated using the formula [26]:

1 $$\eqalign{& SampleSize \cr & = \left[ {Max\left( {Equation{\mkern 1mu} Numberofdimensions} \right)*20} \right] \cr & *\left( {1 + 15\% } \right) \cr} $$

Based on this calculation, the minimum required sample size was 989 participants. The total number of respondents across all four countries was 1,055, meeting the robust SEM requirement.

#### Data collection

The survey was administered to the participants between July and October 2024.

Participants were provided with informed consent at the beginning of the questionnaire, and the survey was distributed through university networks, student forums, and social media platforms. The study employed a double-blind design to minimize bias, ensuring anonymity for survey administrators and respondents.

#### Inclusion and exclusion criteria

The inclusion criteria required participants to be enrolled in undergraduate nursing programs and to have at least basic experience with technology. Students unwilling to provide informed consent were excluded. The study adhered to ethical standards, and approval was obtained from institutional review boards in each participating country.

### Measures

The survey instrument included three main sections: (I) *Socio-demographic data*, (II) *GAIAS*, and (III) *AIAS*. The first section focuses on the socio-demographic data of participants, while the second section evaluates their acceptance of Gen AI, and the third section assesses the anxiety related to AI technologies. The first section collected essential socio-demographic information, such as participants’ age, gender, year of study, previous experience with technology and AI, access to technology, urban or rural residence, attendance in AI-related courses or workshops, and their perceived impact of AI on future nursing practice. This information helps characterize the sample and provides context for their experiences with AI tools.

The second section, the GAIAS—based on the UTAUT model—was developed and validated by [27] to assess participants’ acceptance of Gen AI tools. This section comprised 20 items designed to measure four key dimensions, utilizing a 5-point Likert scale for responses:

- **PE**: Seven items evaluated participants’ perceptions of the usefulness of Gen AI tools in achieving their academic and personal goals.
- **EE**: Five items assessed the perceived ease of learning and using Gen AI tools.
- **FC**: Three items examined the availability of resources and support for Gen AI tools.
- **SI**: Five items evaluated the influence of peers, instructors, and significant others on participants’ intention to use Gen AI tools.

The third section, the AIAS, which assessed participants’ levels of anxiety related to AI technologies, was developed and validated by [28]. It included four subscales:

- **Learning**: Eight items explored participants’ anxiety about understanding and using AI-related tools and technologies. Responses were rated on a 7-point Likert scale from 1 (not anxious) to 7 (extremely anxious).
- **Job Replacement**: Six items assessed concerns about AI replacing human jobs, including potential overreliance and loss of human reasoning skills.
- **Sociotechnical Blindness**: Four items measured fears of AI misuse, malfunctions, and unintended consequences, such as autonomous behavior.
- **AI Configuration**: Three items evaluated participants’ apprehension about humanoid AI systems, including fear and intimidation caused by humanoid robots.

To ensure cultural and linguistic appropriateness for Arabic-speaking nursing students, both the GAIAS and AIAS were subjected to a rigorous adaptation process. This included translation into Arabic by bilingual experts, followed by back-translation into English by an independent translator to ensure conceptual equivalence. An expert panel of nursing faculty and researchers reviewed the translated items to assess cultural relevance, clarity, and content validity. Minor adjustments were made to enhance contextual fit without altering the core meanings of the original items.

This process ensured that the adapted instruments retained their validity and reliability in the new context, allowing for meaningful assessment of participants’ acceptance and anxiety regarding AI technologies in healthcare education (Table 1).

**Table 1:** Reliability and validity (CFA fit Indices) of the generative artificial intelligence acceptance scale (GAIAS) and artificial intelligence anxiety scale (AIAS)

Scale	Subscale	Items	Cronbach’s Alpha (α)
**GAIAS** (Yilmaz et al., 2024)	Performance Expectancy (PE)	7	**0.97**
	Effort Expectancy (EE)	5	**0.97**
	Facilitating Conditions (FC)	3	**0.95**
	Social Influence (SI)	5	**0.97**
**AIAS** (Wang et al., 2022)	Learning Anxiety	8	**0.96**
	Job Replacement Anxiety	6	**0.91**
	Sociotechnical Blindness Anxiety	4	**0.91**
	AI Configuration Anxiety	3	**0.96**

**CFA Model Fit Indices**:

• *GAIAS*: χ²/df = 3.24, CFI = 0.97, TLI = 0.96, RMSEA = 0.06, SRMR = 0.04

• *AIAS*: χ²/df = 2.98, CFI = 0.95, TLI = 0.94, RMSEA = 0.07, SRMR = 0.05

### Model description and path modeling

The SEM framework, implemented using Python’s *semopy* library, enables the analysis of complex relationships between latent variables and their observed indicators. This dual-component approach consists of structural and measurement models, providing robust hypothesis testing and validation of theoretical constructs. The code incorporates a two-part model:

- **Structural Model**: This model specifies the relationships between latent variables, such as how BIU is influenced by PE, EE, FC, SI, and Anxiety. The relationships are expressed as *BIU ~ PE + EE + FC + SI + Anxiety*.
- **Measurement Model**: This model maps observed variables (e.g., PE1, EE1, etc.) to their respective latent constructs (e.g., PE, EE, etc.) using factor loadings.

The implementation involves defining the structural and measurement models, loading the dataset, and estimating path coefficients indices. The code uses the *semopy* library to fit the model, compute path coefficients, R-squared values, and factor loadings. The SEM analysis integrates factor analysis and path analysis to validate the relationships between latent variables while ensuring the reliability and validity of the observed data.

### Data analysis

The collected data were analyzed using IBM Statistical Package for Social Sciences (SPSS) version 27 and Python’s *semopy* library for descriptive and SEM analyses. Descriptive statistics, including frequencies, percentages, means, and standard deviations, were used to summarize the demographic characteristics of the participants (Table 2).

## Results

### Demographic and Technology-Related characteristics

Descriptive statistics, including frequencies, percentages, means, and standard deviations, were used to summarize the demographic characteristics of the participants (Table 2), providing an overview of their sociodemographic profiles. Table 2 shows that Egypt had the highest proportion of female participants (56.1%) and Yemen the lowest (53.2%). Average ages were similar across countries (21.66–22.45 years). Most students in Egypt and Jordan were sophomores, while Yemen had the highest proportion of seniors (69.5%). AI experience was highest in Jordan (75.3%) and Egypt (70.5%), but very low in Yemen (12.9%). Intermediate tech skills were most common. Access to personal computers was lowest in Yemen (7.3%) and Saudi Arabia (9.0%), while smartphone use was highest in Egypt (85.6%) and Jordan (83.6%). Most Egyptian students (59.3%) believed AI would be essential for future nursing, compared to 37.3% in Yemen.

**Table 2:** Demographic and Technology-Related characteristics of participants

Variables		Frequency (Percentage)			
		Egypt	Jordan	Saudi Arabia	Yemen
Gender	Female	175 (56.10%)	158 (55.05%)	223 (100%)	124 (53.22%)
	Male	137 (43.90%)	129 (44.95%)	0 (0%)	109 (46.78%)
Age in Years^*^		22.26$\:\pm\:$3.03	21.66$\:\pm\:$3.98	22.03$\:\pm\:$2.51	22.45$\:\pm\:$2.15
Year of Study	Freshman Sophomore Junior Senior	0.0 (0.00%) 117 (37.50%) 101 (32.40%) 94 (30.01%)	47 (16.37%) 87 (30.31%) 86 (29.96%) 67 (23.36%)	25 (11.20%) 77 (34.50%) 62 (27.80%) 59 (26.40%)	16 (6.90%) 26 (11.20%) 29 (12.40%) 162 (69.50%)
Previous Experience with AI	Yes	220 (70.50%)	216 (75.30%)	139 (62.30%)	30 (12.9%)
	No	92 (29.50%)	71 (24.70%)	84 (37.70%)	203 (87.1%)
Previous Experience with Technology	None Basic	75 (24.0%) 62 (19.9%)	79 (27.50%) 87 (30.30%)	73 (32.70%) 52 (23.30%)	74 (31.80%) 55 (23.60%)
	Intermediate	156 (50.0%)	103 (35.9%)	91 (40.80%)	88 (37.80)
	Advanced	19 (6.1%)	18 (6.3%)	7 (3.10%)	16 (6.90%)
Access to Technology	Personal Computer Smartphone Reliable Internet Access None	40 (12.8%) 267 (85.6%) 0 (0.00%) 5 (1.6%)	46 (16.0%) 240 (83.6%) 0 (0.00%) 1 (3.0%)	20 (9.00%) 42 (18.80%) 127 (57.00%) 34 (15.20%)	17 (7.3%) 12 (5.2%) 174 (74.7%) 30 (12.90%)
Perceived Impact of AI on Future Nursing Practice	AI will be essential	185 (59.30%)	117 (40.8%)	104 (46.6%)	87 (37.30%)
AI will be helpful, but not essential	84 (26.90%)	114 (39.7%)	88 (39.50%)	85 (36.5%)	
AI will have a limited role AI will not be necessary AI will be detrimental	24 (7.70%) 8 (2.60%) 11 (3.50%)	43 (15.0%) 6 (2.10%) 7 (2.40%)	28 (12.60%) 2 (0.90%) 1 (0.40%)	29 (12.40%) 10 (4.30%) 22 (9.40%)	

Note: * represents **mean ± standard deviation** values for age in years

### Demographic influences on acceptance and anxiety toward generative AI in nursing students

Table 3 highlights how demographic characteristics influence nursing students’ acceptance of GenAI across four dimensions. Male students scored significantly higher than females in Performance Expectancy (*p* = 0.005), Facilitating Conditions (*p* = 0.010), and Social Influence (*p* = 0.028). Social Influence was also affected by the year of study, with sophomores reporting the highest scores (*p* = 0.030). Students with prior AI experience had significantly lower Effort Expectancy scores (*p* = 0.005), indicating greater ease of use. Advanced technology experience correlated with higher scores in Facilitating Conditions (*p* = 0.022). Additionally, students who perceived AI as essential for future nursing practice had significantly higher scores across all acceptance dimensions (*p* < 0.001).

**Table 3:** Scores of each dimension and statistical test results under different demographic characteristics for nursing students’ acceptance toward GenAI (*N* = 1,055)

Characteristics									
		Performance Expectancy		Effort Expectancy		Facilitating Conditions		Social Influence	
		Score	t/F, *P*	Score	t/F, *P*	Score	t/F, *P*	Score	t/F, *P*
Gender	Female	27.18 ± 4.99	t =-2.786, P = 0.005^***^	18.68 ± 3.39	t =-1.707, *P* = 0.088	11.22 ± 2.15	t =-2.593, P = 0.010^**^	17.93 ± 3.93	t =-2.201, P = 0.028^*^
	Male	28.03 ± 4.61		19.05 ± 3.34		11.56 ± 1.93		18.45 ± 3.58	
Year of Study	Freshman Sophomore Junior Senior	27.91 ± 4.598 27.97 ± 4.589 27.78 ± 4.960 27.45 ± 4.813	F = 0.766, *P* = 0.513	19.02 ± 3.441 18.98 ± 3.233 19.03 ± 3.409 18.77 ± 3.419	F = 0.414, *P* = 0.743	11.48 ± 2.017 11.58 ± 1.898 11.45 ± 1.975 11.31 ± 2.126	F = 1.069, *P* = 0.361	17.48 ± 3.886 18.66 ± 3.396 18.36 ± 3.717 16.05 ± 3.879	F = 3.001, P = 0.030^*^
Previous Experience with AI	Yes	27.45 ± 4.89	t =-0.292, *P* = 0.771	18.43 ± 3.39	t =-2.807, P = 0.005^***^	11.39 ± 1.99	t = 0.720, *P* = 0.472	18.16 ± 3.70	t = 0.481, *P* = 0.631
	No	27.54 ± 4.67		19.03 ± 3.25		11.30 ± 2.04		18.04 ± 3.79	
Previous Experience with Technology	None Basic	27.59 ± 5.285 28.05 ± 4.250	F = 1.577, *P* = 0.193	18.91 ± 3.716 18.95 ± 3.113	F = 1.697, *P* = 0.166	11.53 ± 2.129 11.57 ± 1.742	F = 3.213, P = 0.022^*^	18.32 ± 3.997 18.38 ± 3.327	F = 0.613, *P* = 0.607
	Intermediate	27.45 ± 4.488		18.76 ± 3.155		11.18 ± 2.029		18.05 ± 3.593	
	Advanced	28.64 ± 5.350		19.87 ± 3.334		11.81 ± 2.313		18.57 ± 4.405	
Access to Technology	Personal Computer Smartphone Reliable Internet Access None	28.03 ± 5.015 27.69 ± 4.692 00.00 ± 00.00 25.00 ± 8.989	F = 1.311, *P* = 0.270	19.34 ± 3.195 18.86 ± 3.361 00.00 ± 00.00 16.17 ± 5.811	F = 3.486, P = 0.031^*^	11.60 ± 2.169 11.42 ± 1.973 00.00 ± 00.00 10.00 ± 3.521	F = 2.047, *P* = 0.130	18.66 ± 3.712 18.21 ± 3.695 00.00 ± 00.00 17.00 ± 5.933	F = 1.308, *P* = 0.271
Perceived Impact of AI on Future Nursing Practice	AI will be essential	29.50 ± 3.788		19.55 ± 3.096		11.91 ± 1.743		19.24 ± 5.015	
	AI will be helpful, but not essential	27.08 ± 4.346	F = 58.345, P < ***0.001*** ^***^	18.85 ± 3.148	F = 19.204, P < ***0.001*** ^***^	11.32 ± 1.972	F = 26.983, P < ***0.001*** ^***^	17.96 ± 5.015	F = 25.856, P < ***0.001*** ^***^
	AI will have a limited role AI will not be necessary AI will be detrimental	25.58 ± 4.963 21.35 ± 7.277 22.73 ± 5.505		18.13 ± 3.366 15.31 ± 5.034 16.71 ± 4.161		10.96 ± 1.940 9.00 ± 3.046 9.88 ± 2.482		16.77 ± 5.015 14.77 ± 5.015 16.02 ± 5.015	

Table 4 explores significant demographic differences in anxiety levels toward GenAI across four domains. Male students reported higher anxiety than females in Learning (*p* = 0.028), Job Replacement (*p* = 0.003), and Sociotechnical Blindness (*p* = 0.007), though no gender difference was observed in AI Configuration (*p* = 0.960). The year of study showed no significant impact on any anxiety dimension (*p* > 0.05). Students with prior AI experience demonstrated slightly higher Sociotechnical Blindness anxiety (*p* = 0.05), while technology familiarity played a key role—those with only basic experience had significantly higher Sociotechnical Blindness anxiety than advanced users (*p* = 0.007). Access to smartphones and reliable internet was associated with lower anxiety in Job Replacement (*p* = 0.025), Sociotechnical Blindness (*p* = 0.006), and AI Configuration (*p* = 0.017). Finally, students who perceived AI as essential to future nursing practice reported significantly higher anxiety in Learning (*p* = 0.002), Job Replacement (*p* = 0.004), and AI Configuration (*p* = 0.014), suggesting a link between AI relevance and anxiety levels.

**Table 4:** Scores of each dimension and statistical test results under different demographic characteristics for nursing students’ anxiety toward GenAI (*N* = 1,055)

Characteristics									
		Learning		Job Replace-ment		Sociotechnical Blindness		AI Configuration	
		Score	t/F, *P*	Score	t/F, *P*	Score	t/F, *P*	Score	t/F, *P*
Gender	Female	24.59 ± 12.76	F = 4.86 P = 0.028*	26.05 ± 12.12	F = 8.81 P = 0.003**	17.21 ± 7.82	F = 7.32 P = 0.007**	11.52 ± 6.12	F = 0.003**, *P* = 0.960
	Male	25.99 ± 11.65		28.65 ± 10.99		19.13 ± 7.09		13.34 ± 6.03	
Year of Study	Freshman Sophomore Junior Senior	25.06 ± 12.32 25.90 ± 12.35 25.78 ± 11.87 25.06 ± 11.96	F = 0.371, *P* = 0.774	28.52 ± 10.77 27.45 ± 11.71 27.60 ± 11.39 27.86 ± 11.52	F = 0.230, *P* = 0.875	19.26 ± 7.037 18.79 ± 7.731 18.06 ± 7.280 18.24 ± 7.347	F = 1.009, *P* = 0.388	13.36 ± 6.265 12.53 ± 293 12.75 ± 6.102 12.63 ± 5.981	F = 0.445, *P* = 0.721
Previous Experience with AI	Yes	25.44 ± 11.89	F = 41, *P* = 0.52	27.74 ± 11.78	F = 1.27, *P* = 0.26	18.59 ± 7.72	F = 3.85, *P* = 0.05	12.73 ± 6.12	F = 0.005** *P* = 0.94
	No	24.82 ± 12.35		27.77 ± 11.43		18.21 ± 7.30		12.47 ± 6.13	
Previous Experience with Technology	None Basic	26.62 ± 11.82 25.76 ± 11.63	F = 2.522, *P* = 0.056	26.87 ± 11.69 29.13 ± 10.99	F = 2.432, *P* = 0.064	17.55 ± 7.317 19.55 ± 7.133	F = 4.012, P = 0.007**	12.35 ± 6.043 13.25 ± 5.953	F = 1.763, *P* = 0.153
	Intermediate	24.32 ± 12.12		27.69 ± 11.288		18.55 ± 7.4440		12.76 ± 6.125	
	Advanced	23.98 ± 15.06		26.23 ± 13.15		18.00 ± 8.777		11.58 ± 7.352	
Access to Technology	Personal Computer Smartphone Reliable Internet Access None	24.52 ± 12.76 25.72 ± 11.95 00.00 ± 00.00 16.00 ± 9.94	F = 2.494, *P* = 0.083	26.03 ± 7.037 28.06 ± 7.731 00.00 ± 00.00 19.17 ± 15.25	F = 3.695, P = 0.025*	17.65 ± 7.951 18.63 ± 7.292 00.00 ± 00.00 10.00 ± 7.772	F = 5.074, P = 0.006**	11.89 ± 6.190 12.86 ± 6.096 00.00 ± 00.00 7.17 ± 5.231	F = 4.100, P = 0.017*
Perceived Impact of AI on Future Nursing Practice	AI will be essential	25.06 ± 12.32	F = 4.239, P = 0.002**	28.52 ± 10.77	F = 3.825, P = 0.004**	19.36 ± 7.03	F = 2.264, *P* = 0.060	13.36 ± 6.26	F = 3.125, P = 0.014*
	AI will be helpful, but not essential	25.90 ± 12.35		27.45 ± 11.71		18.79 ± 7.73		12.53 ± 6.29	
	AI will have a limited role AI will not be necessary AI will be detrimental	25.78 ± 11.87 24.52 ± 11.967 00.00 ± 00.00		27.60 ± 11.39 27.86 ± 11.52 00.00 ± 00.00		18.06 ± 7.28 18.24 ± 7.34 00.00 ± 00.00		12.75 ± 6.10 12.63 ± 5.98 00.00 ± 00.00	

### Variable and correlation analysis

In this study, we set BIU and the key constructs from the TAM—PE and EE—as latent variables. These constructs align with the following subscales and observed variables:

- **PE (PU)**: Seven items measuring students’ perceptions of the benefits of GenAI in achieving academic goals.
- **EE (PEOU)**: Five items assessing the ease of learning and using GenAI tools.
- **FC**: Three items assessing the availability of resources and support for GenAI adoption.
- **SI**: Five items evaluating the impact of peers, instructors, and societal encouragement on AI adoption.
- **Anxiety**: Four subscales representing students’ apprehensions about AI learning, job replacement, sociotechnical blindness, and humanoid AI configuration.

Due to variations in item scoring across scales, mean values were calculated for each observed variable, and the data were normalized prior to performing Pearson correlation analysis. A strong positive correlation (*r* = 0.644 – PE/EE) suggests that students who perceive GenAI as useful also find it easier to use, reinforcing TAM’s theoretical assumptions. Additionally, we conducted a factor analysis on the variables in the measurement model, including the PE and EE scales. The Kaiser-Meyer-Olkin (KMO) values for PE and EE were 0.923 and 0.880, respectively, and Bartlett’s Test of Sphericity was significant for both scales (PE: χ² = 4683.768, df = 21, *p* < 0.001; EE: χ² = 2764.706, df = 10, *p* < 0.001), confirming the data’s suitability for factor analysis. The factor loadings of the observed variables ranged from 0.797 to 0.855 across both scales, all exceeding the 0.7 threshold, indicating strong statistical significance. Detailed results are presented in Table 5. We further performed factor rotation analysis using the maximum variance method. After suppressing coefficients below 0.5, we identified that the observed variables grouped into distinct components corresponding to the latent variables of the model, thereby verifying the feasibility of the measurement model.

**Table 5:** Confirmatory factor analysis for PE/PU and EE/PEOU

Indicators	Mean	Standard Deviation	Factor Loadings	Communalities
PU ≤ Useful in daily life (PE1)	3.925	0.843	0.797	0.635
PU ≤ Increases important chances (PE2)	3.964	0.825	0.835	0.697
PU ≤ Helps get things done faster (PE3)	4.169	0.757	0.815	0.664
PU ≤ Increases productivity (PE4)	3.911	0.844	0.820	0.672
PU ≤ Makes life easier (PE5)	3.998	0.839	0.844	0.712
PU ≤ Useful for daily life (PE6)	3.871	0.849	0.839	0.704
PU ≤ Solves problems (PE7)	3.885	0.841	0.805	0.647
PEOU ≤ Learning how to use (EE1)	3.715	0.856	0.843	0.711
PEOU ≤ Easy to leverage (EE2)	3.941	0.729	0.797	0.635
PEOU ≤ Easy to use (EE3)	3.699	0.851	0.855	0.731
PEOU ≤ Easy to become skilled (EE4)	3.816	0.789	0.818	0.670
PEOU ≤ Clear interaction (EE5)	3.748	0.809	0.844	0.713

*Extraction Method*: Principal Component Analysis

### Parameter Estimation and verification analysis

#### BIU as a mediator

BIU was calculated as a weighted combination of PE (PU), EE (PEOU), FC, SI, and Anxiety using unstandardized and standardized beta coefficients. BI was first computed using the formula:

2 $$\:\:\:\:\:\:\:\:\:\:\:\:\:\:\:\:\:BIU=0.200*(PE+EE+FC+SI+Anxiety)\:\:$$

The unstandardized beta coefficients for each predictor were used to calculate the standardized beta coefficients, which quantify the relative contribution of each factor while accounting for their variability. The results showed that PE had a standardized beta coefficient of 0.307 ($\:{\beta\:}_{PE}$), indicating a moderate positive effect. This suggests that students who believe GenAI tools could enhance their academic performance are likelier to use them. EE had a lower coefficient of 0.217 ($\:{\beta\:}_{EE}$), showing that the ease of use of these tools positively influences students’ intentions to adopt them. FC, such as the availability of resources like internet access and devices, exhibited a relatively small but significant effect, with a standardized beta of 0.129 ($\:{\beta\:}_{FC}$). SI also played a significant role, with a beta coefficient of 0.239 ($\:{\beta\:}_{SI}$), indicating that encouragement from peers or faculty increases students’ likelihood of using AI tools. Interestingly, Anxiety emerged as the most influential factor, with a coefficient of 0.779 ($\:{\beta\:}_{Anxiety}$), demonstrating that emotional factors such as anxiety strongly impact students’ decisions to adopt GenAI tools.

BIU was recalculated using standardized z-scores for each predictor variable to improve interpretability. The z-scores are calculated as follows:

3 $$\:{Z}_{x}=\frac{X-{\mu\:}_{X}}{{\sigma\:}_{X}}$$

where *X* = individual data point, *µ* = mean of the dataset, and σ = standard deviation of the dataset. Using the z-scores, the standardized BIU was then calculated as:

4 $$\eqalign{ BIU = & \left( {\beta {\>_{PE}}\>x\>{Z_{PE}}} \right) + \>\left( {\beta {\>_{EE}}\>x\>{Z_{EE}}} \right) \cr & + \>\left( {\beta {\>_{FC}}\>x\>{Z_{FC}}} \right) + \left( {\beta {\>_{SI}}\>x\>{Z_{SI}}} \right)\cr & + \>\left( {\beta {\>_{Anxiety}}\>x\>{Z_{Anxiety}}} \right) \cr}$$

#### Hypotheses testing and model verification

**Fig. 2:**
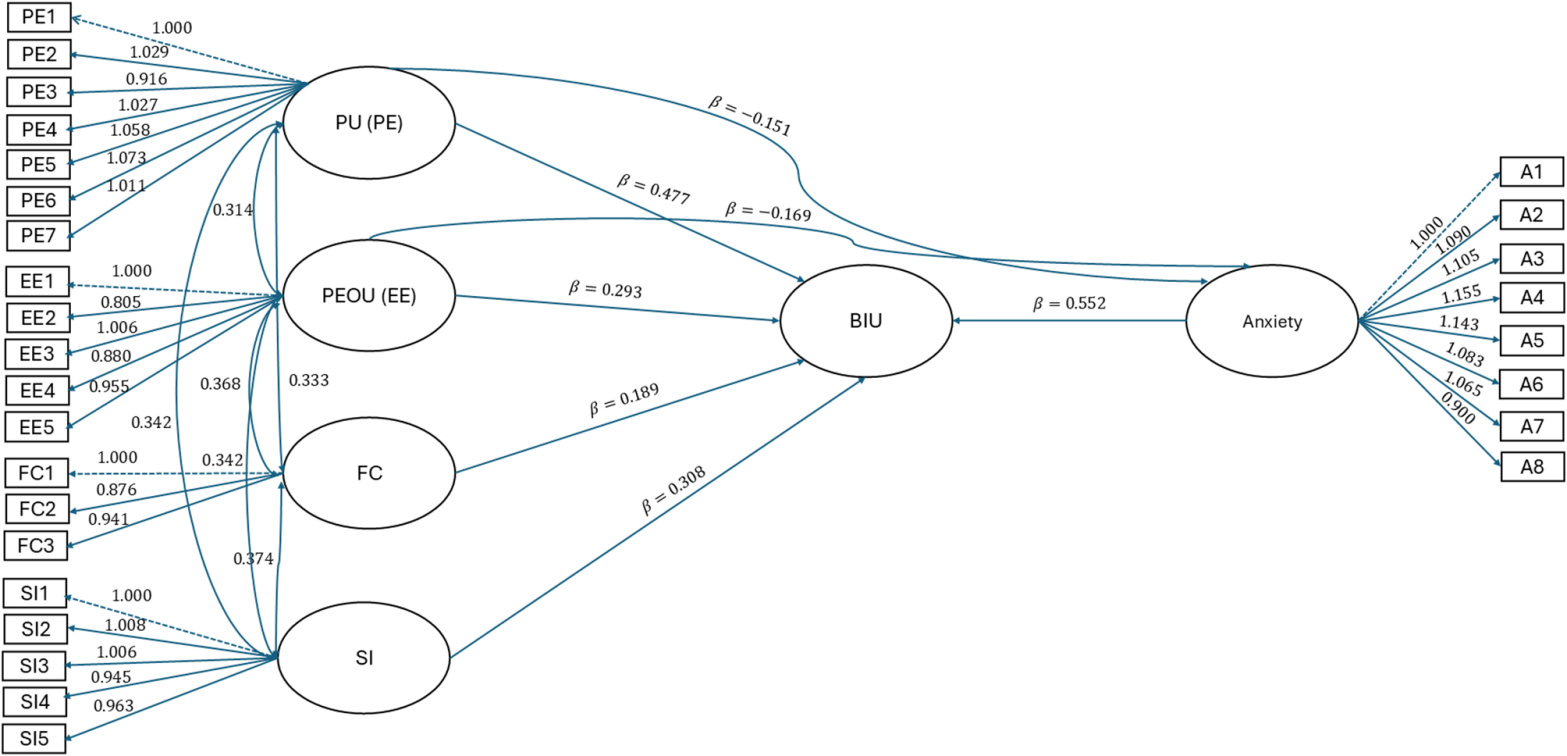
Model fitting results and path coefficients. PU (PE) = Perceived Usefulness (Performance Expectancy), PEOU (EE) = Perceived Ease of Use (Effort Expectancy), FC = Facilitating Conditions, SI = Social Influence, BIU = Behavioral Intention to Use, and Anxiety. The numbers on the arrows represent the standardized path coefficients (β values). Observed variables (e.g., PE1–PE7, EE1–EE5, FC1–FC3, SI1–SI5, A1–A8) indicate measurement items with factor loadings (λ). Correlations among latent variables are also depicted

Python’s *semopy* library was used to fit the SEM, and the results are shown in Fig. 2. The analysis results provide insights into the relationships between latent variables through path coefficients and factor loadings, collectively assessing the model’s performance (Table 6).

Table 6 presents the summary of path coefficients and factor loadings in the structural model. The model verification was analyzed and explained through the path analysis. The relationship between PE and BIU demonstrates a strong and significant positive association, with a path coefficient of 0.477, a z-value of 17.566, and a *p*-value of 0.000, thereby supporting H1. Similarly, the relationship between EE and BIU is significant, with a path coefficient of 0.293, a z-value of 10.667, and a p-value of 0.000, affirming H2. These results highlight the critical roles of usefulness and ease of use in driving behavioral intentions. Anxiety emerges as a significant moderating factor in the model. Its direct effect on BIU is substantial, with a path coefficient of 0.552, a z-value of 33.455, and a *p*-value of 0.000, supporting H3. This highlights anxiety’s influence in moderating the relationships between PE, EE, and BIU.

Additional factors such as FC and SI also contribute meaningfully to the model. FC exhibits a path coefficient of 0.189, a z-value of 4.447, and a *p*-value of 0.0009, indicating a significant positive effect on BIU. This confirms (H4), reinforcing the role of external support systems in fostering AI adoption. SI similarly shows a notable impact, with a path coefficient of 0.308, a z-value of 13.150, and a *p*-value of 0.000, supporting (H5). This highlights the importance of peer and institutional encouragement in shaping behavioral intentions toward AI usage. These findings collectively validate the model’s explanatory power and the relevance of these constructs in influencing behavioural intentions to use GenAI tools.

**Table 6:** Summary of path coefficients, and factor loadings

Variable	Relationship	Estimate ($\:\varvec{\beta\:}$)	Std. Error	z-value	*p*-value	Hypothesis	Loadings
BIU	~ PE		0.027	17.566	< 0.001	H1 Supported	-
BIU	~ EE	0.293	0.027	10.667	< 0.001	H2 Supported	-
BIU	~ FC	0.189	0.043	4.447	0.0009	H4 Supported	-
BIU	~ SI	0.308	0.023	13.150	< 0.001	H5 Supported	-
BIU	~ Anxiety	0.552	0.016	33.455	< 0.001	H3 Supported	-
PE	Loadings	-	-	-	-	-	1.000-1.073
EE	Loadings	-	-	-	-	-	0.804–1.007
FC	Loadings	-	-	-	-	-	0.876- 1.000
SI	Loadings	-	-	-	-	-	0.945–1.008
Anxiety	Loadings	-	-	-	-	-	0.900- 1.156

Table 6 provides insights into patterns observed in the relationships between latent variables and their corresponding indicators. Key observations include:

#### Model Explanatory Power and Effect Sizes

The model’s explanatory ability was evaluated using R^2^ values, which range from 0 to 1. Higher values indicate greater explanatory power. An R^2^ value close to 0.50 suggests moderate explanatory power, while a value close to 0.75 indicates high explanatory power. Table 7 summarizes the explanatory power and effect sizes of the model’s latent variables.

**Table 7:** R^2^ value and f^2^ value

Path	*R* ^2^	Adjusted *R*^2^	f^2^	Effect size
BIU ~ PE	0.7509	0.7432	0.40	Medium
BIU ~ EE			0.30	Medium
BIU ~ FC			0.20	Small
BIU ~ SI			0.25	Medium
BIU ~ Anxiety			0.50	Large

The R^2^ values indicate that the combined effects of PE, EE, FC, SI, and Anxiety explain 75.09% of the variance in BIU. This reflects the model’s high level of explanatory power. The adjusted R^2^ values provide a slightly conservative estimate by accounting for the number of predictors in the model, with a value of 0.7432 across all relationships. This reinforces the robustness of the explanatory ability of the constructs. The f^2^ values, which measure the effect size of exogenous variables on endogenous variables, are categorized as follows:

- **Small effect**: 0.02 < f^2^ ≤ 0.15.
- **Medium effect**: 0.15 < f^2^ ≤ 0.35.
- **Large effect**: f^2^ > 0.35.

From Table 7, it can be observed that:

- Anxiety has the largest effect on BIU (f^2^ = 0.50), indicating a strong influence.
- PE (f^2^ = 0.40), EE (f^2^ = 0.30), and SI (f^2^ = 0.25) exhibit medium effect sizes.
- FC (f^2^ = 0.20) demonstrates a smaller, yet significant, effect size.

These results suggest that the model has strong explanatory power and highlight the varying degrees of influence that each construct exerts on BIU GenAI tools.

## Discussion

The rapid advancement of Gen AI is reshaping healthcare delivery and education. In this context, understanding nursing students’ acceptance, anxiety, and behavioral intentions toward AI is critical, particularly in regions like Middle East, as it can significantly influence the future integration of AI technologies in nursing [29]. This study aimed to examine these factors using a TAM-based SEM approach, focusing on nursing students across four Middle Eastern countries: Egypt, Jordan, Saudi Arabia, and Yemen. By exploring cross-country differences in AI exposure, technology access, and perceptions, this study provides important insights to guide the development of targeted educational strategies that foster equitable AI integration in nursing education and practice.

Our findings show considerable disparities in AI familiarity and access among nursing students. AI exposure was highest in Egypt (70.5%) and Jordan (75.3%), while students in Yemen reported significantly lower experience (12.9%). These variations highlight the influence of socio-economic and infrastructural differences on students’ engagement with GenAI tools. The elevated exposure levels in Egypt and Jordan may reflect more extensive educational initiatives, stronger institutional support for digital learning, and greater national investment in AI development. In contrast, the limited availability of personal computers in Saudi Arabia and Yemen presents a significant barrier to practical engagement with AI technologies, with many students relying primarily on smartphones for digital access. These insights emphasize the importance of implementing context-sensitive educational strategies that address regional disparities and promote equitable access to AI resources and training across diverse learning environments.

Perceptions of AI’s role in nursing were generally positive, with most students recognizing its potential to support future practice. Egyptian students expressed the highest optimism (59.3% viewed AI as essential), whereas a notable proportion of Yemeni students (9.4%) viewed AI as potentially detrimental. The uneven distribution of digital literacy and exposure emphasizes the importance of incorporating structured AI education into nursing programs, particularly in countries facing infrastructural and educational barriers. Correlational analyses support the foundational assumptions of TAM. A strong positive association was observed between PE and EE (*r* = 0.644), consistent with previous TAM studies suggesting that tools perceived as easy to use are more likely to be considered beneficial [30, 31]. EE was also significantly correlated with FC (*r* = 0.704), emphasizing the role of technical resources and support in shaping user perceptions. This is in line with studies that emphasize the role of facilitating conditions in improving technology acceptance [32, 33]. Likewise, the relationship between FC and SI (*r* = 0.692) [34, 35] and between PE and SI (*r* = 0.672) [36, 37]suggests that peer and institutional encouragement significantly shape attitudes toward AI adoption, echoing findings from earlier technology adoption research.

However, the negative associations identified between Anxiety and other TAM constructs, including PE (β = −0.151) and EE (β = −0.169), suggest that higher levels of anxiety may inhibit positive perceptions of AI tools. This is consistent with existing research on AI-related anxiety, which has emphasized the role of emotional factors such as job replacement concerns, sociotechnical blindness, and AI configuration fears in reducing AI self-efficacy [38–40]. The observed moderating influence of Anxiety on both PE/PU and EE/PEOU reinforces the notion that emotional barriers may interfere with the cognitive appraisal of AI tools, particularly in the context of nursing students who often encounter stress when engaging with emerging technologies. Previous studies have also confirmed that anxiety about AI learning and job security can significantly hinder the perceived ease of use and broader acceptance of such tools [41–43]. The findings of this study offer meaningful insights into the key factors shaping nursing students’ BIU GenAI tools. Notably, PE/PU demonstrated a moderate positive effect on BIU (β = 0.477), suggesting that students who recognize the academic benefits of AI tools are more inclined to adopt them. This outcome is consistent with the foundational assumptions of theTAM, which emphasizes perceived usefulness as a primary driver of technology adoption. Likewise, EE/PEOU was found to have a significant positive impact (β = 0.293), highlighting the role of user-friendliness in encouraging acceptance and utilization of new technologies [44–46].

To alleviate AI-related fears and promote positive engagement, several targeted strategies should be considered. First, embedding AI literacy modules within nursing curricula can demystify AI tools and reduce anxiety stemming from a lack of understanding. These modules should focus on practical applications, ethical implications, and human-AI collaboration to build confidence and digital resilience. Second, simulation-based training and faculty-led workshops can serve as safe environments for experimentation and learning, encouraging hands-on familiarity while reducing fear of error. Third, addressing job replacement concerns through curriculum-integrated discussions about AI as an assistive—not replacement—technology can help reframe students’ perspectives and reduce existential anxiety. Lastly, creating peer mentoring programs or digital ambassadors among tech-savvy students could further normalize AI usage and encourage peer-to-peer learning, which has shown promise in reducing technology-related apprehension.

The influence of FC and SI on students’ BIU Gen AI tools was also notable, with standardized beta coefficients of 0.189 and 0.308, respectively. These results are consistent with extended TAM models, such as TAM2 and UTAUT, which recognize the role of environmental and social enablers in shaping technology adoption [47, 48]. Access to necessary resources, infrastructure, and encouragement from peers or instructors significantly bolsters students’ willingness to engage with AI technologies, reinforcing the importance of creating a supportive learning ecosystem. Interestingly, Anxiety emerged as the strongest predictor of BIU, with a standardized beta coefficient of 0.552, indicating a substantial impact. Emotional responses particularly concerns surrounding AI learning, job displacement, and uncertainty can strongly influence students’ willingness to adopt new technologies. This aligns with existing research on AI-related anxiety, emphasizing the need for proactive strategies to mitigate emotional barriers and promote positive engagement with AI in healthcare education [49, 50].

More specifically, the anxiety reported by students may stem from three interrelated sources: learning anxiety, job-replacement anxiety, and ethical or role-related concerns. Learning anxiety refers to students’ fear of not understanding or effectively using AI tools, particularly in low-resource settings where exposure is limited. Job-replacement anxiety arises from uncertainty about AI’s future role in healthcare and fears that automation may reduce the demand for human nurses. Ethical concerns involve questions about accountability, decision-making, and the perceived depersonalization of patient care [51]. Addressing these anxieties requires targeted interventions: AI literacy modules can reduce learning anxiety by enhancing understanding and hands-on skills; faculty-guided discussions that frame AI as a supportive rather than substitutive technology can alleviate job-replacement fears; and ethics-integrated AI education can build confidence around the responsible use of AI in clinical decision-making [52]. By tailoring interventions to the underlying type of anxiety, educators can more effectively foster acceptance and emotional readiness among future nurses.

This study employed SEM approach to examine nursing students’ BIU GenAI tools, yielding meaningful insights into the factors shaping AI acceptance. The path coefficients and factor loadings derived from the SEM analysis confirmed significant associations between latent variables, thereby supporting the proposed hypotheses and aligning with prior literature on technology acceptance in educational contexts [53, 54]. Specifically, the strong positive relationship between PE and BIU (β = 0.477) validates Hypothesis 1, indicating that students who perceive AI tools as academically beneficial are more inclined to adopt them—consistent with the core principles of the TAM [55]. Likewise, the significant effect of EE on BIU (β = 0.293) reinforces Hypothesis 2, emphasizing that ease of interaction plays a crucial role in shaping students’ willingness to engage with AI technologies [56, 57].

The model demonstrated a high explanatory power, with an R² value of 0.7509 for BIU, indicating that PE, EE, FC, SI, and Anxiety collectively explain over 75% of the variance in BIU. This level of explanatory strength is comparable to those found in robust SEM models within educational research. The adjusted R² of 0.7432 further reinforces the stability and reliability of the model. Analysis of effect sizes (f² values) offers deeper insight into the relative contribution of each construct. Anxiety emerged as the most impactful predictor with a large effect size (f² = 0.50), highlighting the critical role of emotional responses in shaping technology adoption—an observation supported by prior research. PE (f² = 0.40), EE (f² = 0.30), and SI (f² = 0.25) showed moderate effect sizes, underscoring their substantial roles in influencing behavioural intention. Although smaller, the effect size for FC (f² = 0.20) was still meaningful, demonstrating the relevance of external support and resource availability in promoting Gen AI acceptance among students.

### Limitations and future directions

This study acknowledges several limitations. One key limitation is the reliance on self-reported survey data, which may introduce response bias. Participants may have been influenced by social desirability or may have misunderstood certain survey questions, potentially affecting the accuracy of their responses. Additionally, the cross-sectional design captures data at a single point in time, limiting the ability to establish causal relationships or observe changes in acceptance, anxiety, or behavioral intention toward GenAI tools over time.

There is also the potential for sampling bias, as students who already have a strong interest in technology or AI may have been more inclined to participate in the survey. This could skew the results toward more favorable attitudes and higher acceptance levels, potentially underrepresenting students who feel indifferent or negatively about AI integration in healthcare.

Looking ahead, longitudinal or interventional research designs are warranted to explore how student attitudes toward GenAI evolve over time and how educational interventions may influence these attitudes. For example, future studies could track actual usage patterns of AI tools before and after targeted training or curriculum changes. Such efforts would help to move beyond intention and better understand real-world adoption and behavioral shifts among nursing students in clinical or educational settings.

### Conclusion

This study provides valuable insights into the factors shaping nursing students’ BIU GenAI tools across four Middle East countries Egypt, Jordan, Saudi Arabia, and Yemen. The findings affirm the central role of PE/PU and EE/PEOU in influencing students’ willingness to adopt AI technologies, aligning with the foundational assumptions of the TAM. The significant positive correlation between PE/PU and EE/PEOU highlights the need for intuitive, user-friendly AI tools that clearly demonstrate academic and clinical value. FC and SI also emerged as meaningful predictors of BIU, emphasizing the importance of technical infrastructure, peer engagement, and faculty support in fostering AI adoption. However, Anxiety was identified as the most impactful factor, acting as a powerful moderator that can dampen students’ confidence and willingness to engage with GenAI tools. In particular, anxiety related to AI learning complexity and fears of job displacement significantly weakened perceived ease of use and usefulness, pointing to the urgent need for strategies that directly address emotional barriers. The cross-country comparison revealed stark disparities in AI exposure and digital resource accessibility. Students in Egypt and Jordan reported higher familiarity with GenAI, likely due to greater institutional investment and digital readiness. Conversely, students in Yemen reported minimal AI experience, and both Yemen and Saudi Arabia exhibited limited access to personal computers and stable internet connectivity. These socio-economic and infrastructural constraints directly impact students’ digital preparedness and readiness to adopt emerging technologies.

To address these challenges and promote equitable AI adoption, the study recommends several targeted educational and institutional strategies:

- **Integrate AI training into nursing curricula**, including hands-on modules and clinical simulations.
- **Develop mobile-friendly learning platforms** to accommodate students with limited computer access.
- **Improve digital infrastructure** and ensure reliable internet connectivity through institutional support.
- **Establish faculty mentorship programs** to foster a culture of encouragement and reduce anxiety through guided learning.
- **Promote digital literacy and self-efficacy**, enabling students to confidently interact with AI tools without fear of failure.

By implementing these strategies, nursing education programs can help students transition smoothly into AI-integrated healthcare environments, ensuring they are equipped with both the technical skills and emotional resilience needed in a rapidly evolving digital landscape.

This study contributes to the growing body of knowledge on GenAI acceptance in nursing education by identifying both key drivers and barriers to adoption. The SEM results validate the proposed hypotheses, demonstrating the substantial impact of PE/PU, EE/PEOU, SI, FC and Anxiety in predicting students’ BIU. Through addressing emotional concerns, improving infrastructure, and implementing targeted training initiatives, nursing programs can better equip students for the evolving digital landscape of healthcare. Future research should adopt longitudinal and m ixed-methods approaches to assess the long-term effectiveness of tailored interventions, and to explore how GenAI competencies develop over time in diverse educational and cultural contexts.

## Data Availability

Data will be available from the authors upon reasonable request.
