# Correction to: Generative artificial intelligence acceptance, anxiety, and behavioral intention in the middle east: a TAM-based structural equation modelling approach

**DOI:** 10.1186/s12912-025-03640-6

**Published:** 2025-08-01

**Authors:** Mona Gamal Mohamed, Polat Goktas, Shimaa Abdelrahim Khalaf, Aycan Kucukkaya, Ibrahim Al-Faouri, Ebtisam Abd Elazeem Saber Seleem, Awatef Ibraheem, Aya M. Abdelhafez, Saleh O. Abdullah, Hanan Nasef Zaki, Abdulqadir J. Nashwan

**Affiliations:** 1 Adult Health Nursing. RAK College of Nursing, RAK Medical and Health Sciences University, Al Qusaidat, Near RAK Hospital, PO Box: 11172, Ras Al-Khaimah, UAE; 2 UCD School of Computer Science, University College Dublin, Belfield, Dublin, Ireland; 3 College of Applied Medical Sciences in Yanbu Governorate, Taibah University, Yanbu, KSA Saudi Arabia; 4 Department of Community Health Nursing, Faculty of Nursing, Assiut University, Assiut, Egypt; 5 Institute of Graduate Studies, Istanbul University-Cerrahpasa, Istanbul, Turkey; 6 Dean of RAK College of Nursing, RAK Medical and Health Sciences University, Ras Al-Khaimah, UAE; 7 Jordan University of Science and Technology, Ar-Ramtha, Jordan; 8 Faculty of Nursing, Zarqa University, Zarqa, Jordan; 9 Nursing Administration Department, Assiut University, Assiut, Egypt; 10 Gynecological and Obstetric Nursing, Faculty of Nursing, Assiut University, Assiut, Egypt; 11 Department of Psychiatric and Mental Health Nursing, Faculty of Medicine & Health Science, Hodeidah University, Al Hudaydah, Yemen; 12 Psychiatric and Mental Health Nursing, Faculty of Nursing, Benha University, Benha, Egypt; 13 Nursing & Midwifery Research Department (NMRD), Hamad Medical Corporation, Doha, Qatar; 14 Department of Public Health, College of Health Sciences, QU Health, Qatar University, Doha, Qatar

**Correction to: BMC Nurs 24, 703 (2025)**.

10.1186/s12912-025-03436-8

Following the publication of the original article [1], it was noted that the author’s name Aycan Kucukkaya was incorrectly written as Aycan Kucukkuya.

The original article [1] has been corrected.
